# Differences in Microbial Communities Stimulated by Malic Acid Have the Potential to Improve Nutrient Absorption and Fruit Quality of Grapes

**DOI:** 10.3389/fmicb.2022.850807

**Published:** 2022-05-19

**Authors:** Peng Si, Wei Shao, Huili Yu, Guoyi Xu, Guoqiang Du

**Affiliations:** ^1^College of Horticulture, Hebei Agricultural University, Baoding, China; ^2^Zhengzhou Fruit Research Institute, Chinese Academy of Agricultural Sciences (CAAS), Zhengzhou, China; ^3^College of Forestry, Henan Agricultural University, Zhengzhou, China

**Keywords:** malic acid, CLPP, Illumina MiSeq sequencing, nutrient absorption, total soluble sugar, titratable acid, *Bacillaceae*, *Woeseiaceae*

## Abstract

Malic acid is a component of the rhizosphere exudate and is vital for crop growth. However, little information is available about the effects of external applications of malic acid on the nutrient absorption and quality of grape fruit, and few studies have been performed on the relationship between the changes in the rhizosphere microbial community and nutrient absorption and fruit quality of grapes after adding malic acid. Here, the LM (low concentration of malic acid) and HM (high concentration of malic acid) treatments comprised 5% and 10% malic acid (the ratio of acid to the total weight of the fertilizer) combined with NPK fertilizer, respectively. Applying malic acid changed the grape rhizosphere microbial community structure and community-level physiological profile (CLPP) significantly, and HM had a positive effect on the utilization of substrates. The microbial community structure in the rhizosphere of the grapes with added malic acid was closely related to the CLPP. The N and P content in the leaves and fruits increased after applying malic acid compared to the control, while K content in the fruits increased significantly. In addition, malic acid significantly reduced the weight per fruit, significantly increased soluble sugar content (SSC) and vitamin C content of the fruit, and significantly improved the fruit sugar-acid ratio and grape tasting score. Moreover, the principal component analysis and grape nutrient and fruit quality scores showed that grape nutrients and fruit quality were significantly affected by malic acid and ranked as 5% malic acid > 10% malic acid > control. Pearson’s correlation heatmap of microbial composition, nutrient absorption and fruit quality of the grapes showed that the grape microbial community was closely related to grape nutrients and fruit quality. Adding malic acid was positively correlated to *Planococcaceae*, *Bacillaceae*, *Woeseiaceae* and *Rhodobacteraceae*. Furthermore, *Planococcaceae*, *Bacillaceae*, *Woeseiaceae* and *Rhodobacteraceae* were closely related to grape nutrient absorption and fruit quality. *Bacillaceae* and *Woeseiaceae* were positively correlated with total soluble sugar, while *Planococcaceae* and *Rhodobacteraceae* were positively correlated with titratable acid. Hence, *Bacillaceae* and *Woeseiaceae* were the key bacteria that played a major role in grape fruit quality and nutrient absorption after applying malic acid water-soluble fertilizer.

## Introduction

Malic acid (2-hydroxybutanedioic acid) is a four-carbon dicarboxylic acid (Dai et al., 2018) used as an enzyme substrate (Bassham and Calvin, 1980; Buser-Suter et al., 1982; Casati et al., 1999; Edwards et al., 2001) and a carrier of carbon-reducing power to transfer carbon and reducing power between the cytoplasm and organelles (Drincovich et al., 2001; Scheibe, 2004; Chen et al., 2019). Malic acid often links various metabolic pathways in different organelles and participates in the regulation of various metabolic reactions in plant cells (Outlaw and Oliver, 1977; Song et al., 2009). Malic acid in leaves is involved in the regulation of stomatal opening and closing, which provides a large number of counter ions to open the stomata and take up K^+^. When the stomata are open, the concentration of malic acid in the guard cells is six times higher than that when the stomata are closed, while the K^+^ concentration increases two to four times (Yao et al., 2020). In particular, malic acid has a biological “phytohormone” effect, which promotes the growth and cold resistance of seedlings (Lou et al., 1993; Lasa et al., 2002; Guo et al., 2017).

Malic acid is a low molecular weight organic acid (LMWOA) that is closely related to soil nutrient content and is a link between carbon and nitrogen metabolism. The difference in the ratio of added nitrate-nitrogen to ammonium-nitrogen causes changes in the type and content of organic acids in the rhizosphere (Lasa et al., 2002; Dong et al., 2004). It is an important energy source for bacterial respiration located in the nodules of nitrogen-fixing bacteria (Driscoll and Finan, 2010). This acid provides most of the carbon skeleton for nitrogen fixation (Rosendahl et al., 1990; Galvez, 2000; Schulze et al., 2002; Vance and Heichel, 2003) and participates in the binding of the oxygen diffusion barrier through an osmotic electrical mechanism (Denison, 1998). Malic acid exchanges and chelates with Fe and Al ligands, thereby reducing the adsorption of P in the soil (Wang et al., 2016), resulting in a larger pool of P in the soil solution that is available for plant uptake (Bolan et al., 1994). Malic acid is secreted by potassium-dissolving bacteria to dissolve potassium from aluminum potassium silicate (Prajapati and Modi, 2012) and drives the surface chemical reactions of acid hydrolysis and complex dissolution and promotes the release of mineral potassium and soil potassium, which increases the effective potassium content in the soil (Wang and Wang, 2009).

As a plant rhizosphere exudate, malic acid has a screening effect on the plant rhizosphere microbial community (Jones, 1998; Rudrappa et al., 2008; Berendsen et al., 2012; Chen et al., 2012; Lakshmanan et al., 2012; Beauregard et al., 2013; Tan et al., 2013; Yuan et al., 2018). When *Arabidopsis* was infected with *Pseudomonas syringae*, the secretion of malic acid into the rhizosphere increased, contributing to the proliferation of *Bacillus subtilis* FB17 in the rhizosphere. Hence, the formation of a *B. subtilis* biofilm is closely related to the presence of malic acid (Berendsen et al., 2012; Chen et al., 2012). *Bacillus amyloliquefaciens* T-5 is significantly induced by malic acid in a chemotactic reaction and cluster movement but has no significant effect on the formation of the biofilm (Tan et al., 2013). Moreover, malic acid, citric acid, and oxalic acid are common rhizosphere exudates of watermelon that induce the biocontrol bacterium *Paenibacillus polymyxa* SQR-21 to drive toward the root. Malic acid has a strong driving ability (Ling et al., 2011).

Therefore, malic acid has the potential to act as a synergist of NPK water-soluble fertilizer. Research on exogenous malic acid has mainly focused on its mitigation effect in response to heavy metal stress (Ebrahimian and Bybordi, 2014; Chen et al., 2020; Yao et al., 2020), and several studies have investigated the preservation of fresh-cut *Lilium* cv. Brunello as well as growth and flowering in *Gazania* and the uptake of K by tobacco (Darandeh and Hadavi, 2012; Talebi et al., 2014; Han et al., 2016). A previous study showed that malic acid combined with NPK fertilizer significantly improved pear fruit quality and nutrient uptake (Shao et al., 2022). However, the role of the microbial community in this process is not clear.

In this study, Shine Muscat grapes were used as experimental materials, conventional NPK fertilizer was used as the control, and 5% and 10% malic acid combined with NPK fertilizer were applied as treatments. We explored the relationship between the grape rhizosphere microbial community, nutrient uptake and fruit quality after adding malic acid to evaluate how malic acid-driven microbial communities affect grape nutrients and fruit quality and the prospect of applying malic acid as a synergist for fruit quality improvement.

## Materials and Methods

### Plant Material and Trial Information

The experiment was carried out in Kangcun, Xinxiang City, Henan Province, China (35°9′28″N, 113°42′17″E) from April to September 2019. The physical and chemical properties of the 0–20 cm soil layers were measured according to Lu (2000) and were as follows: organic matter 0.62%, nitrate-nitrogen 93.25 mg/kg, ammonium-nitrogen 59.20 mg/kg, available P 102.92 mg/kg, available K 213.4 mg/kg, pH 6.7 and electrical conductivity 152.47 us/cm.

Shine Muscat grapes (*Vitis labrusca* × *V. vinifera*) were planted in 2014 and arranged for the trials, and the spacing between the rows was 2.5 m × 1.5 m. Malic acid and NPK fertilizer were formulated into water-soluble fertilizer solutions in the proportions shown in Table 1. The fertilizers were applied at the flowering, young fruit, fruit expansion and 20-days-before-harvest stages during the grape growing period. Each treatment had three repeated plots, and each plot had nine grape trees. Each plot was arranged randomly. The fruits matured, the grapes were sampled after they matured (16 bunches of grapes from each treatment) and were brought back to the laboratory on September 20, 2019, for testing of various indicators. The rhizosphere soil samples were collected 1 month after the last fertilization, and three grape trees in each plot were randomly collected and mixed into one sample. Then, three soil samples from each treatment were processed, and a portion of each sample was dried naturally, with the rest stored at −80°C for the determination of microbial indicators. KNO_3_, urea (NH_4_N_2_O) and KH_2_PO_4_ were supplied by Sinopharm Chemical Reagent Beijing Co., Ltd. (Beijing, China).

**TABLE 1:** Fertilization program for the Shine Muscat grapes (kg/hm^2^/year).

Treatment	NH_4_N_2_O-KH_2_PO_4_-KNO_3_ (kg/hm^2^/year)	Malic acid (kg/hm^2^/year)
Control	130.89–221.89–283.23	0
LM, low concentration of malic acid	130.89–221.89–283.23	33.47
HM, high concentration of malic acid	130.89–221.89–283.23	70.67

### Analysis of the Community-Level Physiological Profile

According to Si et al. (2018), a community-level physiological profile (CLPP) was constructed using the Biolog EcoPlate (Biolog Inc., Hayward, CA, United States). Three soil samples from each treatment were analyzed for the experiment. Briefly, 1 g of soil and 99 mL of 0.85% sterilized NaCl solution were added to an autoclaved triangular flask, and the flask was shaken at 120 rpm for 30 min and then stored at 4°C for 30 min. A total of 150 μL of the resulting suspension was placed in each well, and the mixture was incubated at 25°C for 192 h. Then, the plates were read every 24 h using a Biolog MicroStation TM reader at both 590 and 750 nm (Guanghua et al., 2008) (Biolog Inc.).

The CLPP was constructed using Biolog Ecoplate (Biolog Inc., Hayward, CA, United States). The 120 h data collected during the exponential phase were used to construct the CLPPs for the Shine Muscat grape rhizosphere soil. Principal component analysis (PCA) was used to assess differences relating to the different amounts of malic acid added for the CLPPs, after normalizing the absorbance associated with each substrate (Kolton et al., 2017). Six C source groups were calculated to assess catabolic activity with the different malic acid treatments (Jiang et al., 2013; Wu et al., 2013).

### Measurement of Soil Physicochemical Properties

Nitrate (NO_3_-N) and ammonium (NH_4_-N) were measured according to Lu (2000) by extracting with 1.0 M KCl at a 1:10 soil-to-solution ratio, followed by measurements using an automated discrete analyser (CleverChem 380, DeChem-Tech Inc., Hamburg, Germany) (Si et al., 2018). According to the method described by Lu (2000), available K was extracted in 1 M ammonium acetate using atomic absorption spectrophotometry (AAS; ZEEnit 700P; German Jena Analytical Instrument Co., Ltd., Jena, Germany), and available P was extracted from the soil samples with 0.5 M NaHCO3 (pH 8.5) and measured spectrophotometrically (Tu-1901; Persee Inc., Beijing, China). The pH was measured using a pH meter (DPH-2; ATAGO, Tokyo, Japan) at a 1:2.5 (w/v) ratio of soil to distilled water. The electrical conductivity of the soil was measured using a conductometer (DEC-2; ATAGO) at a 1:5 (w/v) ratio of soil to distilled water (Lu, 2000). Total carbon (TC) and inorganic carbon (IC) in the soil were determined using a carbon and nitrogen analyser (Primacs100, Skalar, Breda, Netherlands). Soil organic matter (SOM) = 1.724 × (TC − IC).

### Measurement of Leaf Photosynthetic Indices

Leaf photosynthesis was measured using a CIRAS-3 instrument (PP systems, Amesbury, MA, United States) at 1 week before harvest.

### Determination of Related Fruit Quality Indices

Soluble solid content (SSC) was measured with a handheld digital refractometer (PR-101, Atago, Tokyo, Japan). Vitamin C (Vc) content was measured using the 2,6-dichlorophenol indophenol method (Cao et al., 2007). Total soluble sugar (TSS) content was determined by the anthrone method (Wang, 2006). Titratable acid (TA) content was determined by the NaOH titration method (Lu, 2000). The solid acid ratio (SAR) was calculated as TSS/TA. The grape tasting score (TS) was evaluated using a 10-point sensory evaluation according to a previous method (Lin et al., 2020).

### Determination of Plant N, P, and K

N, P, and K contents of the leaves and fruits were determined by digestion with H_2_SO_4_-H_2_O_2_ (Lu, 2000). An automatic discontinuous chemical analyser (Clever Chem 380) was used to determine the N and P contents in leaves and fruit, and the K content was determined using an AAS device (AAS ZEEnit 700P, Jena, Germany).

### DNA Extraction and Polymerase Chain Reaction Amplification

The DNA extracted from three independent soil samples served as a template to amplify the 16S rRNA gene and the internal transcribed spacer (ITS) region. The V3-V4 hypervariable region of the bacterial 16S rRNA gene (Wan et al., 2018) was amplified with the primer pairs 338F (5′-ACTCCTACGGGAGGCAGCAG-3′) and 806R (5′-GGACTACHVGGGTWTCTAAT-3′). The fungal-specific primers (Jamil et al., 2020) ITS3F (5′-GATGAAGAACGYAGYRAA-3′) and ITS4R (5′-TCCTCCG CYYATTGATATGC-3′) were employed to amplify the fungal ITS region. Polymerase chain reaction (PCR) amplification of the 16S rRNA gene was performed as follows: initial denaturation at 95°C for 3 min, followed by 27 cycles of denaturing at 95°C for 30 s, annealing at 55°C for 30 s, extension at 72°C for 45 s, a single extension at 72°C for 10 min and ending at 4°C. The PCR mixtures contained 5× TransStart FastPfu buffer 4 μL, 2.5 mM dNTPs 2 μL, forward primer (5 μM) 0.8 μL, reverse primer (5 μM) 0.8 μL, TransStart FastPfu DNA Polymerase 0.4 μL, template DNA 10 ng and ddH2O up to 20 μL. The PCR reactions were performed in triplicate. The PCR product was extracted after 2% agarose gel electrophoresis and purified using the AxyPrep DNA Gel Extraction Kit (Axygen Biosciences, Union City, CA, United States) according to the manufacturer’s instructions and quantified using the Quantus™ Fluorometer (Promega, Madison, WI, United States).

Polymerase chain reaction amplification of the ITS region was performed using the KAPA HiFiHot Start ReadyMix PCR Kit in a GeneAmp PCR System 9700 instrument (Life Technologies, Carlsbad, CA, United States). The PCR reactions were conducted in 25 μL total volume reaction cocktails consisting of 12.5 μL of KAPA HiFi HotStart ReadyMix (2×), 0.25 μmol L-1 of each primer and 10 ng of the DNA template. Amplification was performed with the following thermal profile: 3 min of initial denaturation at 95°C followed by 27 cycles of denaturation at 95°C for 30 s, annealing at 55°C for 30 s, extension at 72°C for 30 s and a final extension at 72°C for 10 min. After purification, the PCR products were quantified using the 2100 Bioanalyses System (Agilent Technologies Inc., Santa Clara, CA, United States) (Mueller et al., 2000) and pooled at equal concentrations.

### Illumina MiSeq Sequencing and Data Analysis

The purified amplicons were pooled in equimolar concentrations and paired-end sequenced on an Illumina MiSeq PE300 platform (Illumina, San Diego, CA, United States) according to the standard protocols of Majorbio Bio-Pharm Technology Co., Ltd. (Shanghai, China). The raw reads were deposited into the NCBI Sequence Read Archive database (Accession Number: PRJNA786655).

The raw 16S rRNA gene and ITS region sequencing reads were demultiplexed and quality-filtered using fastp version 0.20.0 (Chen et al., 2018) and merged with FLASH version 1.2.7 (Magoč and Salzberg, 2011) using the following criteria: (i) the 300 bp reads were truncated at any site receiving an average quality score < 20 over a 50 bp sliding window, and truncated reads < 50 bp were discarded; reads containing ambiguous characters were also discarded; (ii) only overlapping sequences > 10 bp were assembled according to their overlapped sequence. The maximum mismatch ratio of the overlap region was 0.2. Reads that could not be assembled were discarded; (iii) samples were distinguished according to the barcode (Supplementary Table 1) and primers, and the sequence direction was adjusted, the exact barcode was matched and two nucleotide mismatches were used for primer matching.

Operational taxonomic units (OTUs) with a 97% similarity cut-off (Stackebrandt and Goebel, 1999; Edgar, 2013) were clustered using UPARSE version 7.1 (Edgar, 2013) and chimeric sequences were identified and removed. The taxonomy of each representative OTU sequence was analyzed using RDP Classifier version 2.2 (Wang et al., 2007) against 16S rRNA and the ITS database (e.g., Silva v138) with a confidence threshold of 0.7.

### Data Analysis

Experiments were performed using a completely randomized design. Statistical analysis was performed using SPSS Statistics 22 software (SPSS Inc., Chicago, IL, United States). All data are expressed as the mean ± standard error (SE). One-way analysis of variance and Duncan’s test were used to detect differences. A *P*-value < 0.05 was considered significant. The PCA was performed using Canoco 4.5 (Microcomputer Power, Ithaca, NY, United States). Non-metric multidimensional scaling (NMDS) was conducted and a Pearson’s correlation heatmap was produced using an R package.

## Results

### Microbial Community Composition and Metabolism in the Grape Rhizosphere Soil After Adding Malic Acid

**FIGURE 1:**
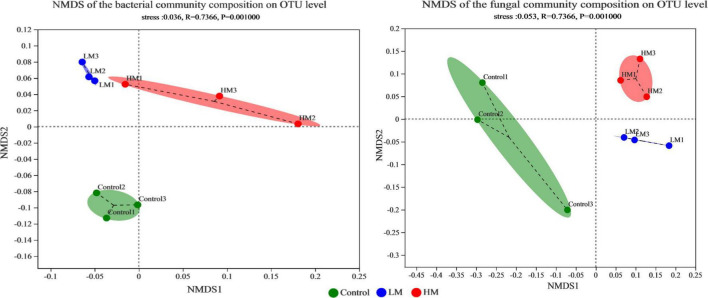
NMDS plots showing differences based on Bray-Curtis distance in the grape rhizosphere microbial community structure after applying different amounts of malic acid. **Left:** bacterial community. **Right:** fungal community.

The number of OTUs in the grape rhizosphere increased, while the OTUs of specific bacteria and fungi decreased after adding malic acid (Supplementary Figure 1). NMDS analysis and the percentage of microbial composition were used to evaluate the effect of applying malic acid on the microbial community structure of the grape rhizosphere (Figure 1 and Supplementary Figure 2). The NMDS of the microbial community revealed that each treatment formed its own cluster, and the control cluster was separated from the malic acid samples (LM and HM clusters). Additionally, the LM cluster was close to the HM cluster in the bacterial and fungal communities. These results demonstrate that the grape rhizosphere microbial community structure changed significantly after applying malic acid.

**FIGURE 2:**
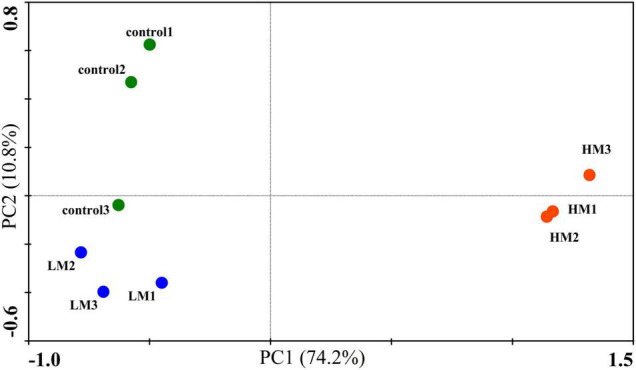
Effect of malic acid on carbon utilization by the grape rhizosphere microbial community. PCA plot of carbon substrate utilization patterns after the addition of malic acid.

The PCA of the soil microbial CLPP showed that the malic acid treatments affected the functional structure of the soil microbial community (Figure 2). Two PCs accounted for 85% of the total variation and each treatment formed its own cluster. The control cluster was close to the LM cluster, and distributed on the negative axis of PC1, whereas the HM cluster was farther away and distributed on the positive axis of PC1. However, this was significantly different from the NMDS analysis of the microbial OTUs by ANOSIM (*p* = 0.001).

The use of six substrate types (polymers, carbohydrates, phenolic compounds, carboxylic acids, amino acids and amines) with different malic acid applications is shown in Supplementary Figure 3. The results show that utilization of the six substrate types by the microbial community changed significantly under the HM treatment (*p* < 0.05). Utilization of the six substrate types by the grape rhizosphere microbial community increased with an increase in the amount of malic acid added. The utilization intensity of HM for the six major carbon sources was significantly higher than that of LM and the control, of which the utilization intensity for phenols was the most significant. Moreover, HM had a positive effect on substrate utilization.

Pearson’s correlation heatmap between bacterial composition and microbial carbon shows that *Gemmatimonadetes* and *Firmicutes* were positively correlated with utilization of the six carbon sources, among which *Gemmatimonadetes*, *Firmicutes* and amines were extremely significantly positively correlated, with correlation coefficients of 0.85 (*p* = 0.003) and 0.81 (*p* = 0.008), respectively (Supplementary Table 2). *Patescibacteria* and *Sumerlaeota* were significantly negatively correlated (*p* < 0.05) with the utilization of the six carbon sources. The correlations between *Patescibacteria* and polymers and amines were −0.7 (*p* = 0.035) and −0.67 (*p* = 0.048), while the correlations between *Sumerlaeota* and phenols, amines, and carbohydrates were −0.68 (*p* = 0.046), −0.7 (*p* = 0.037) and −0.69 (*p* = 0.039), respectively (Supplementary Table 2).

**FIGURE 3:**
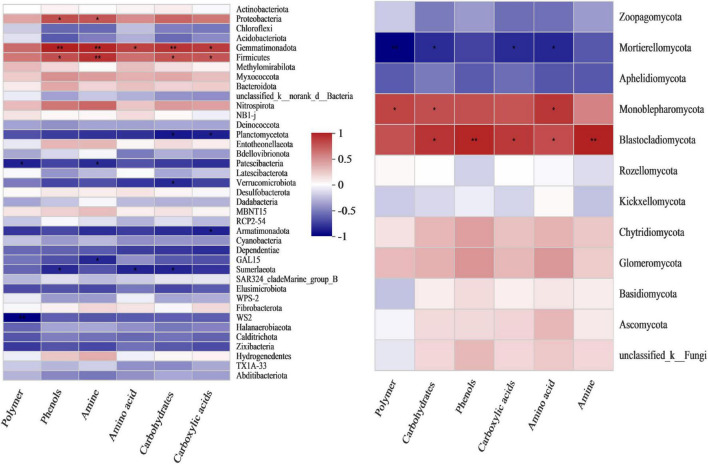
Pearson’s correlation heatmap showing the relationship between microbial composition and metabolism at the phylum level. **Left:** bacterial community. **Right:** fungal community. **Correlation is significant at the 0.01 level. *Correlation is significant at the 0.05 level.

Pearson’s correlation heatmap between fungal composition and microbial carbon metabolism showed that *Mortierellomycota* was negatively correlated with microbial carbon metabolism, and significantly negatively correlated with polymers, carbohydrates, carboxylic acids and amino acids, with correlation coefficients of −0.85 (very significant, *p* = 0.004), −0.7 (*p* = 0.036), −0.71 (*p* = 0.034) and −0.72 (*p* = 0.029), respectively (Figure 3 and Supplementary Table 3). *Monoblepharomycota* was significantly positively correlated with polymers, carbohydrates and amino acids, with coefficients of 0.71 (*p* = 0.033), 0.67 (*p* = 0.048) and 0.76 (*p* = 0.018), respectively (Figure 3 and Supplementary Table 3). *Blastocladiomycota* was very significantly positively correlated with phenols (0.82, *p* = 0.007) and amines (0.84, *p* = 0.005), and significantly positively correlated with carbohydrates (0.77, *p* = 0.016), carboxylic acids (0.75, *p* = 0.02) and amino acids (0.67, *p* = 0.047) (Figure 3 and Supplementary Table 3).

### Nutrient Contents of the Grape Rhizosphere Soil, Leaves and Fruit After Adding Malic Acid

As shown in Table 2, the rhizosphere soil NO3-N trended downward with increased application of malic acid, and that under the LM treatment was significantly lower than the control. In contrast, available P content trended upward, but no significant difference was observed between the treatments. NH4-N content increased first and then decreased, and that under the LM treatment was significantly higher than that under the HM treatment and in the control. No significant difference in available K content was observed among the treatments.

**TABLE 2:** Nutrient contents of the grape rhizosphere soil, leaves, and fruits with different amounts of added malic acid.

Sample Source	Nutrient indicators	Control	LM	HM
Soil (mg/kg)	NO3-N	93.25 ± 5.46a	76.98 ± 2.91ab	61.17 ± 11.55b
	NH4-N	14.8 ± 1.6b	24.13 ± 2.24a	10.62 ± 1.05b
	Available P	102.92 ± 4.47a	117.15 ± 7.76a	119.4 ± 9.34a
	Available K	563.07 ± 22.43a	538.48 ± 17.59a	545.06 ± 16.85a
Leaf (mg/g)	N	21.14 ± 0.15a	22.01 ± 0.34a	21.78 ± 0.22a
	P	1.94 ± 0.05a	2.07 ± 0.07a	2.18 ± 0.14a
	K	8.75 ± 0.25a	9.77 ± 0.23a	8.74 ± 0.82a
Fruit (mg/g)	N	4.85 ± 0.19a	5.46 ± 0.45a	5 ± 0.22a
	P	1.11 ± 0.03a	1.32 ± 0.12a	1.39 ± 0.07a
	K	12.58 ± 0.16b	14.59 ± 0.71a	14.98 ± 0.47a

*Values are presented as the mean ± SE. Different lowercase letters in the same row indicate significant differences (p < 0.05) between treatments with or without the addition of malic acid based on one-way analysis of variance (ANOVA).*

There were no significant differences in grape soil pH between the LM, HM, and control samples. The SOM content and EC value were significantly higher in the LM samples than in the control and HM samples (with SOM being 132.84% higher under LM vs. the control; Supplementary Table 4). The LM and HM treatments significantly increased the net photosynthetic rate (Pn) of leaves. The LM treatment significantly increased stomatal conductivity (Gs) and the water utilization rate (WUE) compared to the control (Supplementary Table 5).

The leaf nutrient analysis showed that although there was no significant difference between the treatments, the N and P contents of the LM and HM leaves (except the K content of HM leaves) tended to be higher than those of the control. In addition, NPK content in the fruit trended upward with increased addition of malic acid, and the K content in the LM and HM fruits was significantly higher than that of the control.

### Grape Fruit Quality After Adding Malic Acid

**FIGURE 4:**
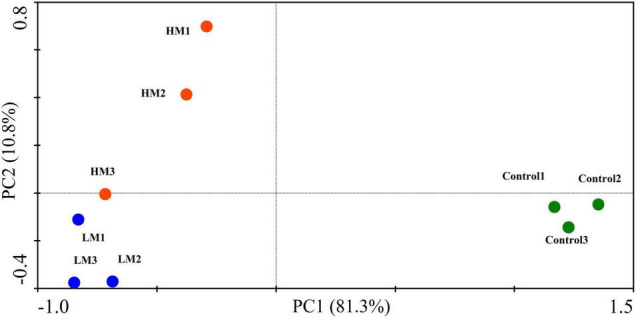
PCA plot showing the effect of adding different amounts of malic acid on grape nutrient absorption and fruit quality.

As shown in Table 3 and Figure 4, the quality of grape fruit was obviously affected by malic acid. Weight per fruit (WPF) decreased significantly as the amount of malic acid added was increased. However, the TSS, Vc, and SSC in the fruit increased significantly after adding malic acid, but no significant differences were observed between LM and HM. In addition, TA of the fruits with HM was significantly higher than that of LM and the control. The solid acid ratio (SAR) and TS of the fruits with LM were significantly higher than those of HM and the control.

**TABLE 3:** Fruit quality of grapes exposed to different amounts of added malic acid.

Fruit quality indicators	Control	LM	HM
Weight per fruit (g)	9.56 ± 0.35a	7.63 ± 0.16b	6.72 ± 0.23c
TSS (%)	12.73 ± 0.25b	22.12 ± 0.53a	21.91 ± 0.13a
Vc (100mg/g)	2.53 ± 0.28b	4.25 ± 0.53a	4.18 ± 0.24a
SSC (%)	18.07 ± 0.31b	21.7 ± 0.92a	22.16 ± 0.81a
TA (%)	0.56 ± 0.01b	0.55 ± 0.03b	0.63 ± 0.01a
Solid-acid ratio	32.08 ± 0.28b	39.48 ± 0.42a	35.02 ± 1.46b
TS	7.48 ± 0.31ab	8.28 ± 0.05a	7.28 ± 0.3b

*Values are presented as the mean ± SE. Different lowercase letters in the same row indicate significant differences (p < 0.05) between treatments with or without the addition of malic acid based on one-way ANOVA.*

As shown in Figure 4, the PCA of nutrient absorption and fruit quality showed that the PC1 and PC2 scores were 81.3% and 10.8%, respectively, and each treatment formed its own cluster. Adding malic acid obviously changed the nutrient absorption and fruit quality of the grapes. The control and malic acid treatments (LM and HM) were located on the positive and negative axes of PC1, respectively. However, the difference in malic acid was mainly reflected in PC2; HM was distributed on the positive axis of PC2, and LM was distributed on the negative axis of PC2. Additionally, the comprehensive score results of grape nutrient absorption and fruit quality showed that LM > HM > control (Table 4).

**TABLE 4:** Comprehensive results of PCA on the effect of adding different amounts of malic acid on nutrient absorption and fruit quality in grape.

Treatment	Principal component score 1	Principal component score 2	Principal component score 3	Principal component score 4	Comprehensive score	Rank
Control 1	−3.31	−0.19	0.05	0.04	−1.94	8.00
Control 2	−2.91	0.28	0.88	0.07	−1.50	7.00
Control 3	−3.54	−0.39	−0.26	0.32	−2.12	9.00
LM1	2.02	0.79	1.24	−0.44	1.44	2.00
LM2	1.80	−1.12	1.67	−1.34	0.92	3.00
LM3	1.63	2.70	0.21	1.20	1.59	1.00
HM1	1.81	−0.49	−0.99	0.95	0.92	4.00
HM2	1.98	−2.38	−0.55	1.00	0.73	5.00
HM3	0.51	0.80	−2.25	−1.79	−0.04	6.00

In summary, although the malic acid treatment reduced WPF, and the high-concentration treatment (HM) risked a reduction in yield, the low-concentration (LM) treatment improved nutrient absorption capacity and fruit quality, resulting in the best taste and the highest nutrient and fruit quality scores.

### Nutrient Absorption and Fruit Quality of the Grapes Were Closely Related to the Rhizosphere Microbial Community

**FIGURE 5:**
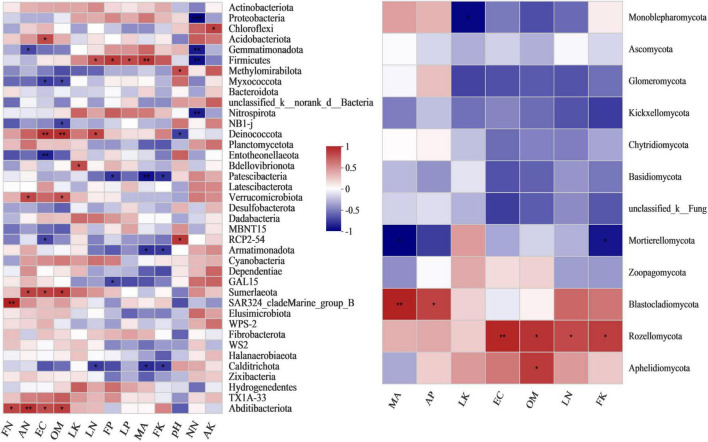
Pearson’s correlation heatmap showing the relationship between microbial composition, environmental factors and nutrient content at the phylum level. **Left:** bacterial community. **Right:** fungal community. MA, malic acid; OM, organic matter; EC, electrical conductivity; LN, leaf nitrogen; LP, leaf phosphorus; LK, leaf potassium; FN, fruit nitrogen; FP, fruit phosphorus; FK, fruit potassium. **Correlation is significant at the 0.01 level. *Correlation is significant at the 0.05 level.

Pearson’s correlation heatmap (Figure 5) shows that the environmental factors of EC, pH, SOM, and added malic acid were closely related to the composition of the bacterial and fungal communities. The amount of added malic acid was very significantly positively correlated with *Firmicutes* and *Blastocladiomycota*, with coefficients of 0.798 (*p* = 0.01) and 0.836 (*p* = 0.005) (Supplementary Tables 6, 7), respectively. In particular, the amount of added malic acid was very significantly negatively correlated with *Patescibacteria*, with a correlation coefficient of −0.87 (*p* = 0.002) (Figure 4 and Supplementary Table 6). Soil OM was significantly positively correlated with *Deinococcota*, *Verrucomicrobiota*, *Sumerlaeota* and *Abditibacteriota*, and had a very significant relationship with *Deinococcota*, with a correlation coefficient of 0.822 (*p* = 0.006), and a significant negative correlation with *Myxococcota* (Figure 4 and Supplementary Table 6). EC had a closer relationship with the bacterial communities than pH and a very significant positive correlation with *Deinococcota*, with a coefficient of 0.808 (*p* = 0.008), and a very significant negative correlation with *Entotheonellaeota*, with a correlation coefficient of −0.815 (*p* = 0.007). The fungus *Rozellomycota* was significantly positively correlated with OM, with a coefficient of 0.775 (*p* = 0.014).

As shown in Figure 5, the bacterial community components were closely related to rhizosphere soil nitrogen content (ammonium-nitrogen and nitrate-nitrogen). In particular, *Proteobacteria*, *Gemmatimonadota*, *Firmicutes* and *Nitrospirota* were very significantly negatively correlated with nitrate-nitrogen content, with coefficients of −0.934 (*p* = 0), −0.837 (*p* = 0.005), −0.881 (*p* = 0.002) and −0.829 (*p* = 0.006), respectively (Supplementary Table 6). Studies on the nutrient and microbial community composition of grape leaves and fruits have shown that grapes are closely related to absorption of the nutrient element potassium. Leaf K content was negatively correlated with *Monoblepharomycota* (Supplementary Table 7, *r* = −0.754, and *p* = 0.019) and positively correlated with *Bdellovibrionota*, with a correlation coefficient of 0.703 (*p* = 0.035). Fruit K content was negatively correlated with *Patescibacteria* and *Calditrichota*, with correlation coefficients of −0.764 (*p* = 0.017) and −0.738 (*p* = 0.023), respectively.

**FIGURE 6:**
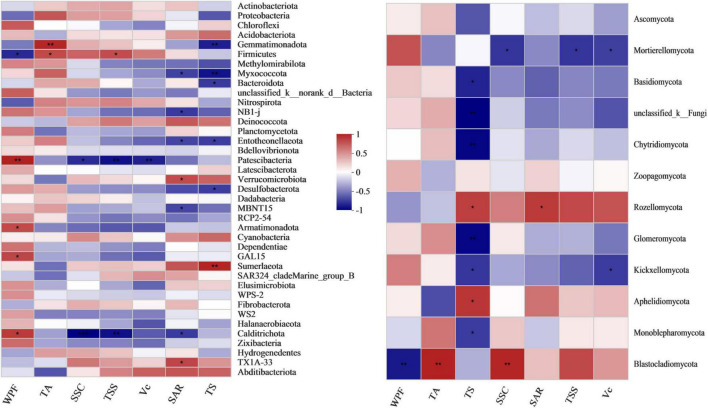
Pearson’s correlation heatmap showing the relationship between microbial composition and grape fruit quality at the phylum level. WPF, weight per fruit; SSC, soluble solid content; Vc, vitamin C; TSS, total soluble sugar; TA, titratable acid; TS, grape tasting score; SAR, solid-acid ratio (TSS/TA). **Correlation is significant at the 0.01 level. *Correlation is significant at the 0.05 level.

Pearson’s correlation heatmap analysis of the fruit quality bacterial community showed that the SAR, TS and WPF were closely related to the bacterial community (Figure 6 and Supplementary Table 8). In particular, TS was closely related to the bacterial and fungal communities, with a very significant negative correlation with *Gemmatimonadota* and *Myxococcota*, and correlation coefficients of −0.82 (*p* = 0.007) and −0.863 (*p* = 0.003), respectively, in the bacterial community, and a very significant positive correlation with *Sumerlaeota*, with a correlation coefficient of 0.836 (*p* = 0.005). TS was very significantly negatively correlated with *Chytridiomycota* and *Glomeromycota* in the fungal community with correlation coefficients of −0.86 (*p* = 0.003) and −0.86 (*p* = 0.003), respectively.

Firmicutes had a significant positive correlation with TA and TSS and a significant negative correlation with WPF (Figure 6). *Patescibacteria* was negatively correlated with TSS, SSC and Vc, with correlation coefficients of −0.867 (*p* = 0.002), −0.773 (*p* = 0.015) and −0.813 (*p* = 0.008), respectively, and a very significant positive correlation with WPF, with a correlation coefficient of 0.847 (*p* = 0.004). *Calditrichota* was significantly negatively correlated with TSS and SAR (*r* = −0.872 and −0.723, *p* = 0.002 and 0.028, respectively), and significantly positively correlated with WPF (*r* = 0.748, *p* = 0.02).

The increase in the ratio of the fungi *Mortierellomycota* and *Blastocladiomycota* reduced fruit quality. *Mortierellomycota* had a significant negative correlation with TSS, SSC and Vc, while *Blastocladiomycota* had a very significant positive correlation with TA, and a very significant negative correlation with WPF, with correlation coefficients of 0.814 (*p* = 0.008) and −0.802 (*p* = 0.009), respectively. The increase in the *Rozellomycota* ratio had the potential to improve fruit quality, which was significantly positively correlated with SAR and TS.

**FIGURE 7:**
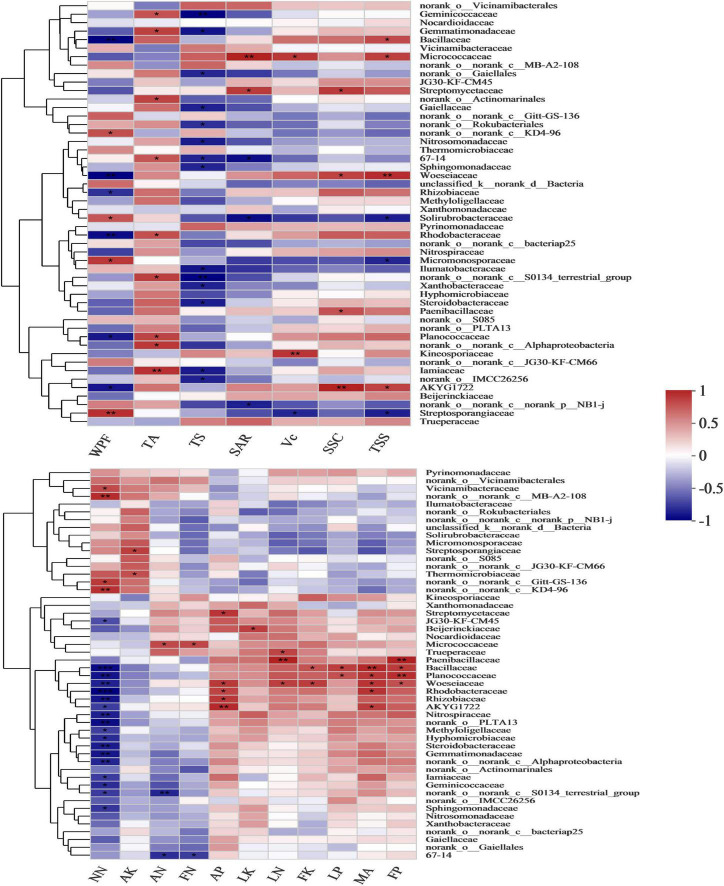
Pearson’s correlation heatmap showing the relationships between bacterial composition, fruit quality and nutrient content at the family level. MA, malic acid; OM, organic matter; EC, electrical conductivity; LN, leaf nitrogen; LP, leaf phosphorus; LK, leaf potassium; FN, fruit nitrogen; FP, fruit phosphorus; FK, fruit potassium; WPF, weight per fruit; SSC, soluble solid content; Vc, vitamin C; TSS, total soluble sugar; TA, titratable acid; TS, grape tasting score; SAR, solid-acid ratio. **Correlation is significant at the 0.01 level. *Correlation is significant at the 0.05 level.

The nutrient absorption and fruit quality of the grapes were significantly affected by the bacterial community at the family level (Figure 7 and Supplementary Tables 8, 9). Adding malic acid was significantly positively correlated with *Planococcaceae* (*p* = 0.008), *Bacillaceae* (*p* = 0.047), *Woeseiaceae* (*p* = 0.012) and *Rhodobacteraceae* (*p* = 0.01), and the correlation coefficients were 0.78, 0.84, 0.79 and 0.8, respectively. In contrast to the malic acid-added treatments, nitrate-nitrogen was extremely significantly negatively correlated with *Planococcaceae*, *Bacillaceae*, *Woeseiaceae* and *Rhodobacteraceae* with correlation coefficients of −0.88 (*p* = 0.002), −0.9 (*p* = 0.001), −0.82 (*p* = 0.006) and −0.95 (*p* = 0), respectively. Similar to the malic acid-added treatments, the P contents of leaves and fruits were significantly positively correlated with *Planococcaceae* (*r* = 0.68 and 0.81, and *p* = 0.045 and 0.008, respectively) and *Bacillaceae* (*r* = 0.75 and 0.77, and *p* = 0.021 and 0.016), and soil available P content was significantly positively correlated with *Woeseiaceae* (*r* = 0.77, and *p* = 0.015) and *Rhodobacteraceae* (*r* = 0.73, and *p* = 0.026). Fruit K content was significantly positively correlated with *Bacillaceae* (*r* = 0.67, and *p* = 0.049) and *Woeseiaceae* (*r* = 0.76, and *p* = 0.018). Therefore, *Planococcaceae*, *Bacillaceae*, *Woeseiaceae* and *Rhodobacteraceae* were related to the malic acid treatments and played an important role in the nutrient absorption of grapes.

Furthermore, contrary to malic acid, *Planococcaceae*, *Bacillaceae*, *Woeseiaceae* and *Rhodobacteraceae* all had significant negative correlations with WPF, with correlation coefficients of −0.75 (*p* = 0.019), −0.84 (*p* = 0.005), −0.84 (*p* = 0.005) and −0.8 (*p* = 0.009), respectively. *Bacillaceae* and *Woeseiaceae* were significantly positively correlated with TSS, with correlation coefficients of 0.72 (*p* = 0.028) and 0.84 (*p* = 0.005), while *Planococcaceae* and *Rhodobacteraceae* were significantly positively correlated with TA (*r* = 0.75 and 0.71, *p* = 0.021 and 0.033, respectively), and the proliferation of *Bacillaceae* and *Woeseiaceae* increased the TSS of fruit exposed to added malic acid.

## Discussion

As an important intermediate product of many metabolic processes in plants, malic acid links multiple metabolic pathways in cells (Fernie and Martinoia, 2009) and plays an important physiological function during plant growth. In addition, malic acid, as one of the main exudates of the plant rhizosphere, affects the composition of the rhizosphere microbial community and soil nutrient cycling.

### The Microbial Community in the Grape Rhizosphere Was Altered by Malic Acid

Adding malic acid affected the pH of the soil, which, in turn, affected the soil microbial community. In addition, it served as a carbon source to stimulate and screen the soil microbial communities. However, the effects of 5% and 10% malic acid combined with NPK fertilizer on soil pH were not significant, and this was consistent with research on peach and pear rhizosphere soil after adding malic acid (Shao et al., 2022). Therefore, malic acid as a carbon source stimulates and screens the soil microbial community, which is the main reason why it affected the grape rhizosphere microbial community.

Malic acid, a major organic acid in root plant secretions, is selectively secreted and effectively signaled to beneficial rhizosphere bacteria, regulating root metabolites during the recruitment of beneficial microorganisms, which emphasizes the breadth and sophistication of plant-microbe interactions (Rudrappa et al., 2008). The secretion of malic acid into the rhizosphere of three emergent plant species has a significant negative correlation with ammonia-oxidizing bacterial activity (Chen et al., 2021). Malic acid induces a stronger chemotactic response and swarming motility of *Bacillus amyloliquefaciens* than citric acid, succinic acid or fumaric acid, which produces a variety of antibiotics with broad-spectrum activity against different plant pathogens, thereby inducing plant host system resistance (Basi et al., 2006; Chen et al., 2009; Arrebola et al., 2010; Ramarathnam et al., 2010; Tan et al., 2013). Additionally, malic acid in the presence of a pathogen recruits the beneficial bacterium *Bacillus subtilis* FB17 to Arabidopsis roots (Rudrappa et al., 2008). Similarly, we revealed that the amount of added malic acid was extremely significantly positively correlated with *Bacillaceae* in the grape rhizosphere. Furthermore, malic acid and citric acid in watermelon root exudates, which are intermediate products of the tricarboxylic acid (TCA) cycle, i.e., also significantly induce *Paenibacillus polymyxa* SQR-21 motility (Ling et al., 2011). Hence, malic acid, as the main organic acid in the rhizosphere exudate, is the second most preferred carbon source for organisms, such as *Bacillus subtilis* and *Azospirillum brasilense* (Bashan and de-Bashan, 2002; Meyer et al., 2011; Rekha et al., 2018). Malic acid and citric acid released from tomato roots attract *Pseudomonas fluorescens* strains (Weert et al., 2002; Liu et al., 2020).

The bacterial community was more sensitive to malic acid than the fungal community. In structuring rhizosphere microbial communities with different root exudates, differences in fungal community structure have been attributed to citric acid and differences in bacterial community structure have been attributed to cisaconitic, citric and malic acids (Dennis et al., 2010).

### Grape Fruit Nutrient Absorption Capacity and Fruit Quality Improve in the Presence of Malic Acid

Soil OM is a dynamic nutrient storage medium that provides macronutrients to produce protein in plants through soil biota activities (Garcia-Pausas and Paterson, 2011; Wood et al., 2018). A low malic acid treatment stimulates the microorganisms and primes the soil organic carbon in a nutrient-poor system (Chowdhury et al., 2014). Hence, the SOM content was significantly increased by 5% malic acid compared with the control.

Malic acid was negatively correlated with NO3-N in the emergent plant rhizosphere of a constructed wetland in northern China (Tan et al., 2013). As the amount of malic acid added increased in the current study, the NO3-N content in the grape rhizosphere decreased and was significantly reduced by 10% malic acid combined with the NPK fertilizer. Additionally, absorption of NH4-N was closely related to ammonium-nitrogen content. Soil NH4-N content increased significantly after adding 5% malic acid combined with the NPK fertilizer. The absorption of NH4-N upregulates the synthesis of malic acid and oxaloacetic acid by promoting the activities of malate dehydrogenase and phosphoenolpyruvate carboxykinase (Britto and Kronzucker, 2005; Wang et al., 2021). Thus, root cytosol alkalinization induced by NH4-N uptake distinctly enhanced the activities of phosphoenolpyruvate carboxylase and malate dehydrogenase but reduced malic enzyme activities (Xu et al., 2021).

Malic acid, as one of the LMWOAs, increases plant-available P fractions by solubilizing inorganic P fractions, which are virtually insoluble, retarding the reaction of fertilizer P with soil components and decreasing the relative saturation of metal ions in solution (Harrold and Tabatabai, 2006; Pavinato et al., 2008; Miller and Fox, 2011; Oral and Uygur, 2018). In addition, plants produce a series of protective mechanisms when exposed to a phosphorus deficiency by secreting small molecules, such as malic acid, into the rhizosphere (Ozawa et al., 1995; Ascencio, 1997; McGrail et al., 2021). Although the ability of malic acid to complex with metal ions is weaker than that of dicarboxylic acid and TCA (McGrail et al., 2021), malic acid combined with NPK fertilizer increased soil available phosphorus content, thereby increasing the phosphorus content of leaves and fruits, and ultimately increasing the absorption of phosphorus by grapes.

K is an essential macronutrient for plant growth that plays important roles in various metabolic processes involving protein synthesis, photosynthesis, enzymes and resistance to pests and diseases (Prajapati and Modi, 2012). Potassium is solubilized from potassium-aluminum silicate minerals through the secretion of different organic acids, such as malic acid and citric acid, by potassium-dissolving bacteria (Prajapati and Modi, 2012). Although no significant difference was observed in the results, the available K content of the grape rhizosphere soil with added malic acid was lower than that of the control, while leaf K content in the 5% malic acid treatment was higher than that in the other treatments. However, regardless of the 5% and 10% malic acid combined with the NPK fertilizer, the K content of fruits was significantly higher than that of the control, indicating that malic acid promoted the absorption of potassium by grapes and contributed to the accumulation of potassium in fruits. Secretion of malic acid into the rhizosphere is strongly affected by potassium status (Freeman, 1967). Moreover, the combination of potassium nutrition and exogenous organic acids improves the absorption of iron by monocots and dicots and mediates iron-biofortified crops (Awad-Allah and Elsokkary, 2020).

Malic acid is stored in vacuoles, constituting a major carbon pool and a potential substrate for respiration (Blanke and Lenz, 1989), but is also the predominant organic acid associated with taste, flavor and juice quality in fruit (Yao et al., 2020). Malic acid promotes plant growth by increasing chlorophyll content and mitigating stress damage to photosynthetic structures, thereby significantly increasing plant biomass (Chen et al., 2020). Photosynthetic assimilates are mainly used for fruit growth during the early stage of fruit development, and the sugar in the fruit accumulates 2 weeks after fruit expansion stops, leading to an increase in SSC (Long et al., 2006). Adding malic acid potentially improved the photosynthetic capacity of grape leaves. We speculate that the photosynthetic rate and water use efficiency of grape leaves would increase after adding malic acid, which facilitates the accumulation of soluble solids in the fruit. Similar results were found in pears when applying malic acid combined with NPK (Shao et al., 2022). A study of organic acids and potassium fertilizer in fruits reported that applying potassium fertilizer increases TA of fruits, particularly malic acid content (Cummings and Reeves, 1971; Du, 1985; Biaiłczyk and Lechowski, 1989), and malic acid content is usually positively correlated with ash alkalinity during fruit ripening, while ash content alkalinity is closely related to potassium content (Genevois and Peynaud, 1947; Souty et al., 1967; Lobit et al., 2006).

### Changes in the Rhizosphere Microbial Community Stimulated by Malic Acid Affect Nutrient Absorption and Fruit Quality of Grapes

Malic acid as a rhizosphere exudate secreted by plants drives microorganisms to participate in OM mineralization that indirectly mediates nutrient uptake and indirectly mediates nutrient absorption through dissolution and chelation of nutrients (McGrail et al., 2021). Malic acid was significantly positively correlated with *Planococcaceae*, *Bacillaceae*, *Woeseiaceae* and *Rhodobacteracea*.

It was revealed that the 5% malic acid treatment increased soil ammonium-nitrogen content and decreased soil nitrate-nitrogen content, while excessive malic acid reduced the available nitrogen content in the soil. However, most ammonia-oxidizing bacteria OTUs were negatively correlated with malic acid content (Fang et al., 2019). Contrary to adding malic acid, nitrate-nitrogen was significantly negatively correlated with *Planococcaceae*, *Bacillaceae*, *Woeseiaceae* and *Rhodobacteraceae*. *Planococcaceae* is composed mainly of the genus *Planococcus*, and *Planococcus* includes denitrifying bacteria (Chen et al., 2016; Ismail et al., 2021). *Bacillaceae*, particularly the genus *Bacillus*, is involved in denitrification and dissimilatory nitrogen reduction to ammonium in several strains, and various members of *Bacillus* have flexible physiological functions during the process of dissimilated nitrate reduction and its intermediates or by-products (Verbaendert, 2014). *Woeseiaceae* is an abundant core member of the microbial community in global marine sediments that are involved in the incomplete denitrification pathway, including subunits of nitrite reduction (nirS) and NO reduction (norB) to the ozone-depleting greenhouse gas N2O (Hinger et al., 2019). However, *Rhodobacteraceae*, which oxidize NH4-N to nitrate or nitrite, is significantly negatively correlated with nitrate-nitrogen (Liu et al., 2018).

Similar to the added malic acid treatments, the P content of leaves and fruits was significantly positively correlated with *Planococcaceae* and *Bacillaceae*, and soil available P content was significantly positively correlated with *Woeseiaceae* and *Rhodobacteraceae*. *B. subtilis*, isolated from mangrove soil in Chollangi, East Godavari, exhibits a phosphate solubilizing ability in the range of 80–100 mg/l (Anzuay et al., 2015). Research on the role of P limitations in shaping soil bacterial communities has revealed that *Firmicutes*, including *Planococcaceae* and *Bacillaceae*, are enriched in high P soils, and *Planococcaceae* is relatively more abundant than *Bacillaceae* (Oliverio et al., 2020). Notably, the abundance of *Planococcaceae* and *Bacillacea* related to functions of carbon degradation and P cycling increase sugarcane yield (Silva et al., 2021). *Rhodobacteraceae* is a family in *Alphaproteobacteria* that is involved in C, N and S cycling processes in the marine environment (Zheng et al., 2015; Zhang et al., 2019). Non-marine *Rhodobacteriaceae* gained high-affinity transporters in response to much lower sulfate concentrations and lost genes associated with reduced sodium chloride and organohalogen concentrations in their habitats (Simon et al., 2017). The bacterial carbon-phosphorus lyase pathway, an enzyme complex that evolved to extract phosphate from phosphonates, is prevalent in a considerable proportion of *Rhodobacteraceae* bacteria (11–40% of organisms) across all ocean regions in the mesopelagic zone (Sosa et al., 2019).

Fruit K content was significantly positively correlated with *Bacillaceae* and *Woeseiaceae*. *Bacillaceae* is a family of potassium-dissolving bacteria (KSB) microorganism that secrete organic acids from insoluble potassium-containing minerals that directly dissolve rock K or chelated silicon (Meena et al., 2014, 2016; Zhang and Kong, 2014). Hence, both the malic acid and the increase in the abundance of *Bacillaceae* with added malic acid stimulated the absorption of potassium by grapes; thus, the proliferation of *Planococcaceae*, *Bacillaceae*, *Woeseiaceae* and *Rhodobacteraceae* stimulated by malic acid has the potential to enhance nutrient absorption of grapes. *Planococcaceae*, *Bacillaceae*, *Woeseiaceae* and *Rhodobacteraceae* were significantly negatively correlated with WPF. *Bacillaceae* and *Woeseiaceae* were significantly positively correlated with TSS, while *Planococcaceae* and *Rhodobacteraceae* were significantly positively correlated with TA. However, SSC and TSS of grape fruit increased after adding malic acid. In addition, the nutrient content of leaves and fruits also increased after the malic acid treatment. *Bacillaceae*, involved in plant rhizosphere growth, and *Woeseiaceae*, involved in the nitrogen cycle, have the potential to improve fruit quality. Therefore, *Bacillaceae* and *Woeseiaceae* were the key bacteria playing a major role in grape fruit quality and nutrient absorption after applying the malic acid water-soluble fertilizer.

## Conclusion

Nutrient absorption and fruit quality of grapes were improved after adding malic acid, and the best formula was 5% malic acid combined with NPK fertilizer. In addition, the structure and carbon metabolism of the soil microbial community were affected significantly by applying malic acid, and the composition of the microbial community was closely related to nutrient absorption and the quality of the grapes. Adding malic acid was significantly positively correlated with *Planococcaceae*, *Bacillaceae*, *Woeseiaceae*, and *Rhodobacteraceae* with correlation coefficients of 0.78, 0.84, 0.79 and 0.8, respectively. The proliferation of *Planococcaceae*, *Bacillaceae*, *Woeseiaceae*, and *Rhodobacteraceae* stimulated by malic acid has the potential to enhance nutrient absorption of grapes. *Bacillaceae* and *Woeseiaceae* were significantly positively correlated with the TSS of grape fruit with correlation coefficients of 0.72 and 0.84, respectively, while *Planococcaceae* and *Rhodobacteraceae* were significantly positively correlated with the TA content of grape fruit (0.75 and 0.71, respectively). Hence, *Bacillaceae* and *Woeseiaceae* are the key bacteria that play a major role in grape fruit quality and nutrient absorption after applying malic acid water-soluble fertilizer.

## Data Availability Statement

The datasets presented in this study can be found in online repositories. The names of the repository/repositories and accession number(s) can be found in the article/Supplementary Material.

## Author Contributions

WS, GX, and HY: conceptualization. WS and GX: methodology. WS: software, formal analysis, and data curation. PS, WS, HY, GX, and GD: validation. WS and HY: investigation. PS: resources and visualization. WS and PS: writing—original draft preparation and writing—review and editing. PS and GD: supervision, project administration, and funding acquisition. All authors have read and agreed to the published version of the manuscript.

## Conflict of Interest

The authors declare that the research was conducted in the absence of any commercial or financial relationships that could be construed as a potential conflict of interest.

## Publisher’s Note

All claims expressed in this article are solely those of the authors and do not necessarily represent those of their affiliated organizations, or those of the publisher, the editors and the reviewers. Any product that may be evaluated in this article, or claim that may be made by its manufacturer, is not guaranteed or endorsed by the publisher.
