# Supplementary figures and images for: Differences in Microbial Communities Stimulated by Malic Acid Have the Potential to Improve Nutrient Absorption and Fruit Quality of Grapes
Source: Front Microbiol. 2022 May 19;13:850807. doi: 10.3389/fmicb.2022.850807 (PMC9159917; doi:10.3389/fmicb.2022.850807)

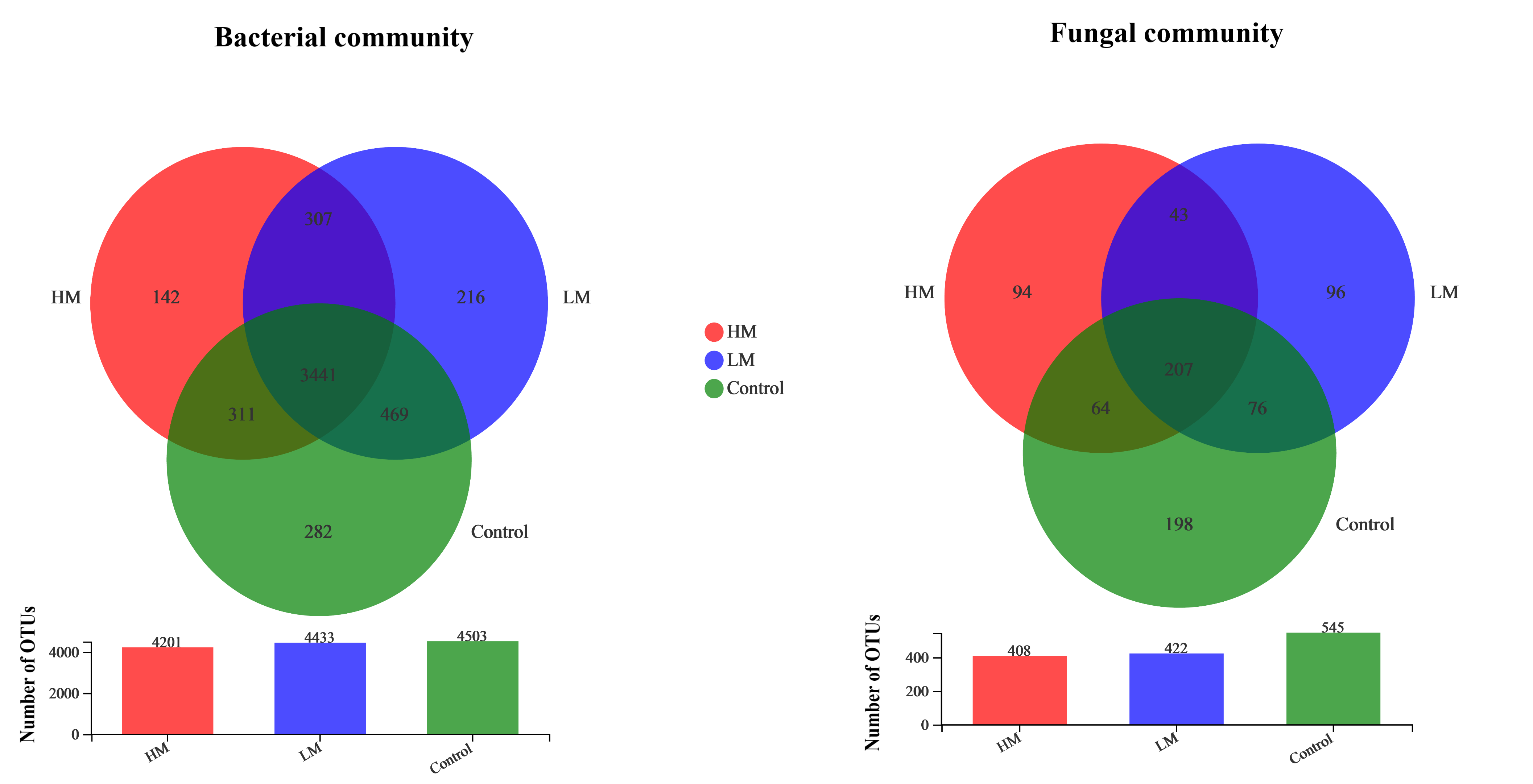

Supplement: Supplementary file 1 [file Image_1.PNG]

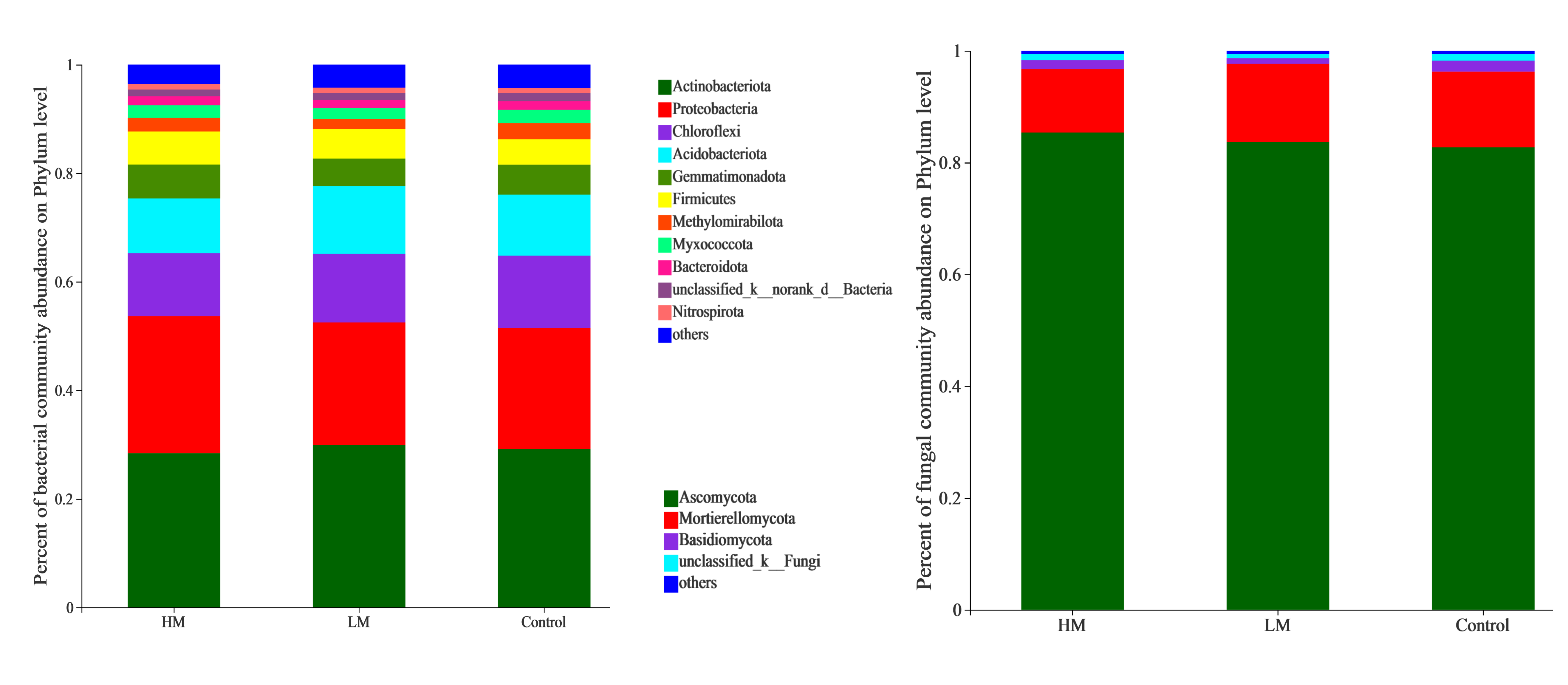

Supplement: Supplementary file 2 [file Image_2.PNG]

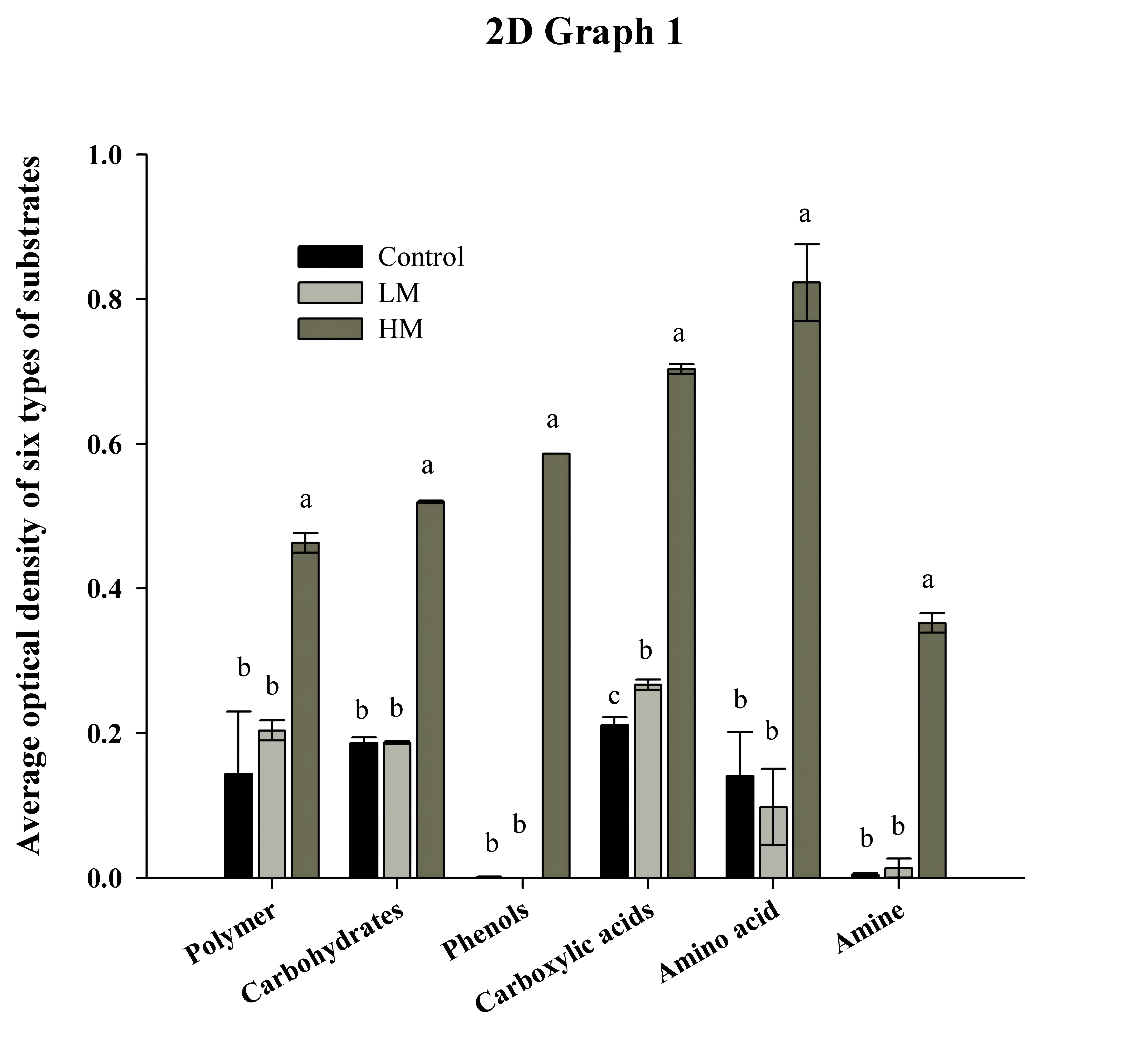

Supplement: Supplementary file 3 [file Image_3.PNG]
